# Ground-state cooling of a mechanical oscillator in a hybrid optomechanical system including an atomic ensemble

**DOI:** 10.1038/s41598-017-16956-4

**Published:** 2017-12-08

**Authors:** Wei Zeng, Wenjie Nie, Ling Li, Aixi Chen

**Affiliations:** 1 Department of Applied Physics, East China Jiaotong University, Nanchang, 330013 China; 2 Department of physics, Zhejiang Sci-Tech University, Hangzhou, 310018 China

## Abstract

We investigate dynamical properties and the ground-state cooling of a mechanical oscillator in an optomechanical system coupling with an atomic ensemble. In this hybrid optomechanical system, an atomic ensemble which consists of two-level atoms couples with the cavity field. Here we consider the case where the atomic ensemble is in higher excitation. Studies show that the atom-field coupling strength can obviously influence the cooling process, and we can achieve the ground-state cooling of the mechanical oscillator by choosing the appropriate physical parameters of the system. Our cooling mechanism has potential applications in quantum information processing and procession measurement.

## Introduction

With the development of quantum optomechanical techniques, more and more attentions focus on the studies of the cavity optomechanics and its application^1–5^. Cooling of the mechanical oscillator to its quantum ground-state is the pivotal step for potential applications such as the exploration of quantum-classical boundary^6–9^ quantum information processing^10–13^ and precision control and measurement^14–19^ and so on. An inevitable limit of the cooling mechanical oscillator to quantum ground-state is thermal noise of the mechanical motion. Thus, a variety of ways in theory or experiment to overcome this problem have been proposed, such as cooling with dissipative coupling^20–25^ quadratic coupling^26^, single-phonon strong coupling^27^, hybrid systems^28,29^ laser pulse modulations^30–34^ and dissipation modulations^35^. Physically, the best way to achieve cooling a mechanical oscillator to quantum ground-state is that both the position and the momentum of the mechanical oscillator quickly tend to 1/2^36^. This means that the effective damping rate of mechanical oscillator should increase significantly^37^, which can be achieved by enhancing the cooling anti-Stokes process and suppressing the heating Stokes’. At the same time, the oscillation frequency of the mechanical motion should be larger than the decay rate of the optical field, so that the sideband condition can be satisfied. Generally speaking, It is difficult to be achieved in most systems except for a few optomechanical systems^38,39^.

In this work, we propose a hybrid optomechanical system, in which the ground-state cooling of the mechanical oscillator is investigated. This system consists of a Fabry-Férot cavity and an atomic ensemble, where each atom in the cavity is treated as a two-level system. In addition, we consider that there exists a higher-order excitation in the two-level atomic ensemble. We derive the expressions of the effective frequency *ω*_*eff*_ and the effective damping rate *γ*_*eff*_ for the movable mirror, which depend on the averaged atom-field coupling strength and the atomical excitation number. We also discuss in detail the cooling characteristics of the mechanical oscillator by counting the effective phonon number. It is found that the atom-field coupling strength and the excitation number have an important influence on the atomical effective damping rate of the mechanical oscillator. In particular, when the low-excitation condition is slightly broken for the atomic ensemble, a large driving strength and a small atom detuning can contribute to enhance the optomechanical coupling and the ground-state cooling.

The paper is organized as follow: In Sec. II, we introduce the system and the Hamiltonian of the system is given. In Sec. III, the equation of mechanical oscillator is derived. The cooling characteristics of the mechanical oscillator are discussed in detail in Sec. IV and the conclusion is presented in Sec. V.

## Model and Theory

**Figure 1:**
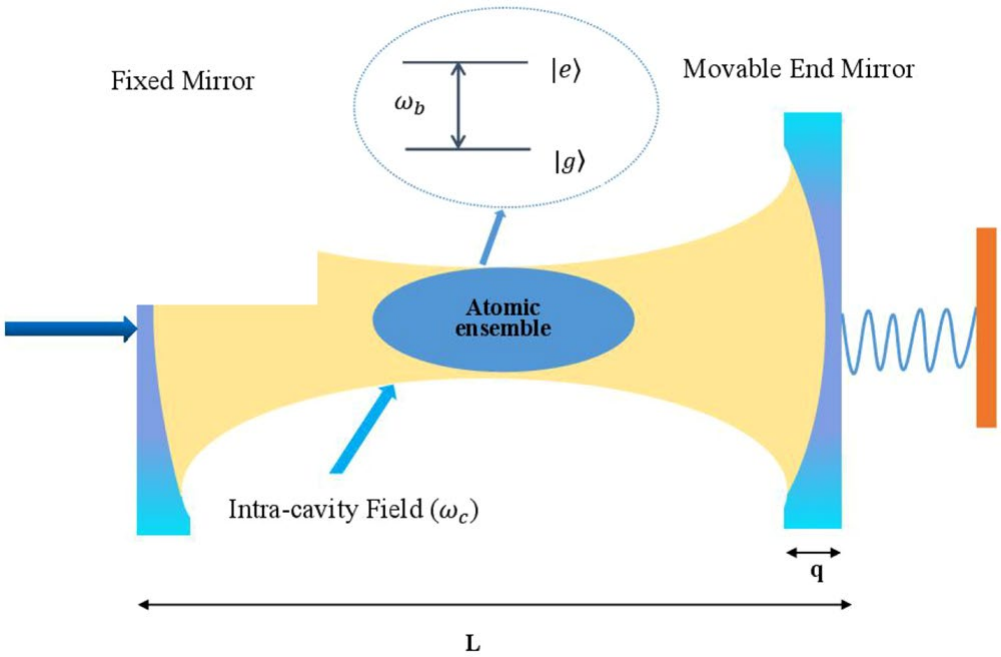
Schematic of the setup studied in the paper. An atomic ensemble are placed in a typical optomechanical cavity with a movable mirror. The cavity mode is driven by a strong input coupling laser field through the left cavity mirror.

We consider a hybrid setup including a typical optomechanical system and an atomic ensemble which consists of lots of atom with two-level energy (see Fig. 1). The ground state $|g\rangle$ and the excited state $|e\rangle$ may correspond, respectively, to states $6{s}^{\mathrm{2\ 1}}{S}_{0}$ and $6s6{p}^{\mathrm{\ 3}}{P}_{1}$ of barium atom^40,41^. The quadrupole transition wavelength between the two states and the decay rate of the excited state to the ground state are λ = 791 nm and λ_*b*_ = 791 kHz, respectively^40^. The intracavity field with frequency *ω*_*c*_ is driven by a control field with frequency *ω*_*f*_ and amplitude *ε*_*c*_. For simplification, we assume that the intracavity photon leakage occurs significantly through the fixed wall and the decay rate of the cavity is *k*. It is noted that in a realistic optical cavity, the decay of the cavity photon can be occurred not only through the fixed but also the movable mirror^42–44^. Further, the movable mirror is regarded as a quantum-mechanical harmonic oscillator with resonance frequency *ω*_*m*_, effective mass *m* and damping rate *γ*_*m*_. Consequently, The total Hamiltonian of the system can be written as^28,45,46^.

1 $$\begin{array}{c}H=\hslash {\omega }_{c}{a}^{\dagger }a+\frac{\hslash {\omega }_{b}}{2}\sum _{i\mathrm{=1}}^{N}{\sigma }_{z}^{(i)}+\frac{{p}^{2}}{2m}+\frac{m{\omega }_{m}^{2}{q}^{2}}{2}-\hslash {G}_{0}{a}^{\dagger }aq\\ +(\hslash ga\sum _{i=1}^{N}{\sigma }_{+}^{(i)}+H\mathrm{.}c\mathrm{.)}+i\hslash ({\varepsilon }_{c}{a}^{\dagger }{e}^{-i{\omega }_{f}t}-H\mathrm{.}c\mathrm{.),}\end{array}$$

where the first term is the energy of the intracavity field; ${a}^{\dagger }(a)$ is the creation (annihilation) operator of the cavity mode satisfying the commutation relation $[a,{a}^{\dagger }]=1$. The second term represents the energy of the atomic ensemble and ${\sigma }_{z}^{(i)}=|e{\rangle }^{(i)(i)}{\langle e|-|g\rangle }^{(i)(i)}\langle g|$, where the ground state and the excited state of the *i* th two-level atom are expressed as $|g{\rangle }^{(i)}$ and $|e{\rangle }^{(i)}$, respectively. The commutation relations for the pseudospin-1/2 operators ${\sigma }_{+}^{i}=|e{\rangle }^{(i)(i)}\langle g|$ and ${\sigma }_{-}^{(i)}=|g{\rangle }^{(i)(i)}\langle e|$ are $[{\sigma }_{+}^{(i)},{\sigma }_{-}^{(i)}]={\sigma }_{z}^{(i)}$ and $[{\sigma }_{z}^{(i)},{\sigma }_{\pm }^{(i)}]=\pm 2{\sigma }_{\pm }^{(i)}$, respectively. The third and fourth terms are the kinetic energy and potential energy of the movable mirror with position operator *q* and momentum operator *p* satisfying the commutation relation $[q,p]=i\hslash$. The last term in the first line describes the optomechanical coupling between the cavity mode and the movable mirror with ${G}_{0}={\omega }_{c}/L$ being the coupling strength^47^, and *L* is the length of the cavity. The first term in the second line gives the interaction between the atomic ensemble and the cavity mode, where *g* represents the averaged atom-field coupling strength^28,48^. The last term in the total Hamiltonian describes the interaction between the cavity field and the coupling field with the amplitude ${\varepsilon }_{c}=\sqrt{\frac{2\kappa P}{\hslash {\omega }_{c}}}$, and *P* is the laser power.

Most of the previous works focus on the low-excitation limit, i.e., $\langle {B}^{\dagger }B\rangle /N\ll 1$ for the atomic ensemble with a large *N*, where *B* and ${B}^{\dagger }$ are the collective annihilation and creation operators of the atoms satisfying $[B,{B}^{\dagger }]=1$ ^49^, defined by the Holstein-Primakoff transformation ${\sum }_{i\mathrm{=1}}^{N}\,{\sigma }_{+}^{(i)}=\sqrt{N}{B}^{\dagger }$ and ${\sum }_{i\mathrm{=1}}^{N}\,{\sigma }_{-}^{(i)}=\sqrt{N}B$. In the present system, we assume that the atomic low-excitation condition is broken so that the higher-order excitation of the atoms is included^50,51^ i.e., ${\sum }_{i\mathrm{=1}}^{N}\,{\sigma }_{+}^{(i)}=\sqrt{N}{B}^{\dagger }(1-\frac{{B}^{\dagger }B}{2N}),$ ${\sum }_{i\mathrm{=1}}^{N}\,{\sigma }_{-}^{(i)}=\sqrt{N}(1-\frac{{B}^{\dagger }B}{2N})B$ and ${\sum }_{i\mathrm{=1}}^{N}\,{\sigma }_{z}^{(i)}\mathrm{=2}{B}^{\dagger }B-N\mathrm{.}$ Then, in the interaction picture with respect to ${H}_{0}=\hslash {\omega }_{f}({a}^{\dagger }a+{B}^{\dagger }B)$, the Hamiltonian of the total system becomes:

2 $$\begin{array}{c}H=\hslash {{\rm{\Delta }}}_{a}{a}^{\dagger }a+\hslash {{\rm{\Delta }}}_{b}{B}^{\dagger }B+\frac{\hslash {\omega }_{m}{p}^{2}}{2}+\frac{\hslash {\omega }_{m}{q}^{2}}{2}-\hslash {G^{\prime} }_{0}{a}^{\dagger }aq\\ +(\hslash Ga{B}^{\dagger }-\frac{\hslash Ga{B}^{\dagger 2}B}{2N}+H\mathrm{.}c\mathrm{.})+i\hslash {\varepsilon }_{c}({a}^{\dagger }-a),\end{array}$$

where $G=g\sqrt{N}$ is the collective coupling strength between the atomic ensemble and the cavity field. ${{\rm{\Delta }}}_{a}={\omega }_{c}-{\omega }_{m}$, ${{\rm{\Delta }}}_{b}={\omega }_{b}-{\omega }_{f}$ and $\delta ={\omega }_{p}-{\omega }_{f}$ are the detuning. The position operator and momentum operator *q* and *p* have been nondimensionalized as $\sqrt{\frac{m{\omega }_{m}}{\hslash }}q\to q$ and $\sqrt{\frac{1}{\hslash m{\omega }_{m}}}p\to p$ in Eq. (2), so the coupling strengths become ${G}_{0}^{\prime} =\sqrt{\frac{\hslash }{m{\omega }_{m}}}{G}_{0}$. In Eq. (2), a constant term $\frac{N{\omega }_{b}}{N}$ has been neglected.

## Dynamical Properties of System

Here we mainly consider the cooling characteristics of the mechanical oscillator. In order to analyze the system in detail, we add Brownian noise and photon losses in the cavity to the present system. Based on the Hamiltonian in Eq. (2), we can obtained the following Heisenberg-Langevin equation^42^:

3 $$\dot{q}={\omega }_{m}\,p,$$

4 $$\dot{p}=-{\omega }_{m}q+{G^{\prime} }_{0}{a}^{\dagger }a-{\gamma }_{m}p+\xi (t),$$

5 $$\dot{a}=-(\kappa +i{{\rm{\Delta }}}_{a})a-iGB+\frac{iG}{2N}{B}^{\dagger }{B}^{2}+iG{^{\prime} }_{0}aq+{\varepsilon }_{c}+\sqrt{2\kappa }{a}_{in},$$

6 $$\dot{B}=-({\gamma }_{b}+i{{\rm{\Delta }}}_{b})B+\frac{iGa{B}^{\dagger }B}{N}-iGa+\frac{iG{a}^{\dagger }{B}^{2}}{2N}+\sqrt{2{\gamma }_{b}}{B}_{in},$$

where *a*_*in*_ and *B*_*in*_ are the input vacuum noise operators with the mean value. They satisfy the correlation functions $\langle {a}_{in}(t){a}_{in}^{\dagger }(t^{\prime} )\rangle =\delta (t-t^{\prime} )$ and $\langle {B}_{in}(t){B}_{in}^{\dagger }(t^{\prime} )\rangle =\delta (t-t^{\prime} )$, respectively^52^. $\xi (t)$ is the Brownian stochastic force with zero mean value, and its correlation function is described by $\langle \xi (t)\xi (t^{\prime} )\rangle =\frac{{\gamma }_{m}}{{\omega }_{m}}\int \frac{d\omega }{2\pi }{e}^{-i\omega (t-t^{\prime} )}\omega [\coth (\frac{\hslash \omega }{2{k}_{B}T})+1]$ ^53^, where *k*_*B*_ is the Boltzmann constant and *T* is the temperature of the reservoir related to the movable mirror.

Based on the Eqs (3–6), we can obtain the steady-state expectation of physical quantities of the system by letting all time derivatives be equal to 0. In the case of the steady-state, values of physical quantities can be calculated as

7 $${p}_{s}=\mathrm{0,}$$

8 $${q}_{s}=\frac{{G^{\prime} }_{0}|{a}_{s}{|}^{2}}{{\omega }_{m}},$$

9 $${a}_{s}=\frac{{\varepsilon }_{c}-iG\sqrt{N}(1-\frac{|{b}_{s}{|}^{2}}{2}){b}_{s}}{\kappa +i{\rm{\Delta }}},$$

10 $$0=-({\gamma }_{b}+i{{\rm{\Delta }}}_{b}){b}_{s}+ig{a}_{s}(|{b}_{s}{|}^{2}-1)+\frac{ig{a}_{s}^{\ast }{b}_{s}^{2}}{2},$$

where ${\rm{\Delta }}={{\rm{\Delta }}}_{a}-{G}_{0}^{\prime} {q}_{s}$ and ${b}_{s}={B}_{s}/\sqrt{N}=m+ni$, *m* and *n* are the real and imaginary part of the steady-state value for the atomic ensemble. As we know, each operator can be written as a linear sum of the steady-state mean value and a small fluctuation, i.e., $O={O}_{s}+\delta O(O=a,B,p,q)$. Inserting the linearized form of the operator *O* into Eqs (3–6), the dynamical equations of the quantum fluctuations in the system can be written as

11 $$\delta \dot{q}={\omega }_{m}\delta p,$$

12 $$\delta \dot{p}=-{\omega }_{m}\delta q+{G}_{0}^{^{\prime} }({a}_{s}^{\ast }\delta a+{a}_{s}\delta {a}^{\dagger })-{\gamma }_{m}\delta p+\xi (t),$$

13 $$\delta \dot{a}=-(\kappa +i{\rm{\Delta }})\delta a+i{G}_{0}^{^{\prime} }{a}_{s}\delta q-i{G}_{1}\delta B+i{G}_{3}\delta {B}^{\dagger }+\sqrt{2\,\kappa }{a}_{in},$$

14 $$\delta \dot{B}=-({\gamma }_{b}+i{\Delta }_{b}^{0})\delta B-i{G}_{1}\delta a+i{G}_{2}\delta {B}^{\dagger }+i{G}_{3}\delta {a}^{\dagger }+\sqrt{2\,{\gamma }_{b}}{B}_{in},$$

where ${G}_{1}=G(1-|{b}_{s}{|}^{2}),\,{G}_{2}=\frac{G{a}_{s}{b}_{s}}{\sqrt{N}},\,{G}_{3}=\frac{G{b}_{s}^{2}}{2}$ and ${{\rm{\Delta }}}_{b}^{0}={{\rm{\Delta }}}_{b}-G({a}_{s}{b}_{s}^{\ast }+{a}_{s}^{\ast }{b}_{s})/\sqrt{N}$. In the process of derivation of Eqs (11–14), we have assumed that there are a large number of the photon in the cavity, i.e., $|{a}_{s}|\gg 1$, so that all the higher terms (*δoδo*) in Eqs (3–6) are neglected. Furthermore, we define new quadrature fluctuation operators for the nonlinear optomechanical system $\delta X=(\delta a+\delta {a}^{\dagger })/\sqrt{2}$, $\delta Y=(\delta a-\delta {a}^{\dagger })/\sqrt{2}i$, $\delta U=(\delta B+\delta {B}^{\dagger })/\sqrt{2}$, $\delta V=(\delta B-\delta {B}^{\dagger })/\sqrt{2}i$ and the corresponding Hermitian input noise operators $\delta {X}_{in}=(\delta {a}_{in}+\delta {a}_{in,\dagger })/\sqrt{2}$, $\delta {Y}_{in}=(\delta {a}_{in}-\delta {a}_{in,\dagger })/\sqrt{2}i$, $\delta u=(\delta {B}_{in}+\delta {B}_{in,\dagger })/\sqrt{2}$, $\delta v=(\delta {B}_{in}-\delta {B}_{in,\dagger })/\sqrt{2}i$. Then, the linearized quantum Langevin equations for the fluctuation operators can be expressed as a more compact form, $\dot{f}(t)=Jf(t)+n(t)$, where ${f}^{T}(t)=(\delta p(t),\delta q(t),\delta X(t),\delta Y(t),\delta U(t),\delta V(t))$ is the column vector of fluctuation operator and ${n}^{T}(t)=\mathrm{(0,}\,\xi ,\sqrt{2\kappa }\delta {X}_{in},\sqrt{2\kappa }\delta {Y}_{in},\sqrt{2{{\rm{\Gamma }}}_{a}}\delta u,\sqrt{2{{\rm{\Gamma }}}_{a}}\delta v)$ is the corresponding column vector of noise. *J* is the drift matrix, which is given by

$${\bf{J}}=(\begin{array}{cccccc}0 & {\omega }_{m} & 0 & 0 & 0 & 0\\ -{\omega }_{m} & -{\gamma }_{m} & {\chi }_{1} & {\chi }_{2} & 0 & 0\\ -{\chi }_{2} & 0 & -\kappa  & {\rm{\Delta }} & -{\alpha }_{2} & {\alpha }_{5}\\ {\chi }_{1} & 0 & -{\rm{\Delta }} & -\kappa  & {\alpha }_{6} & {\alpha }_{2}\\ 0 & 0 & -{\alpha }_{2} & {\alpha }_{5} & -{\gamma }_{b1} & {{\rm{\Delta }}}_{1}\\ 0 & 0 & {\alpha }_{6} & {\alpha }_{2} & -{{\rm{\Delta }}}_{2} & -{\gamma }_{b2}\end{array}),$$

where $${\alpha }_{1}=({G}_{3}+{G}_{3}^{\ast }\mathrm{)/2,}\,{\alpha }_{2}=({G}_{3}-{G}_{3}^{\ast }\mathrm{)/2}i,\,{\alpha }_{3}=({G}_{2}+{G}_{2}^{\ast }\mathrm{)/2}$$, $${\alpha }_{4}=({G}_{2}-{G}_{2}^{\ast }\mathrm{)/2}i,{\alpha }_{5}={G}_{1}+{\alpha }_{1},\,{\alpha }_{6}=$$ $${\alpha }_{1}-{G}_{1}$$, $${\chi }_{1}={G^{\prime} }_{0}\frac{{a}_{s}+{a}_{s}^{\ast }}{\sqrt{2}},{\chi }_{2}={G^{\prime} }_{0}\frac{{a}_{s}-{a}_{s}^{\ast }}{\sqrt{2}i}$$, $${\gamma }_{b1}={\gamma }_{b}+{\alpha }_{4},{\gamma }_{b2}={\gamma }_{b}-{\alpha }_{4},{{\rm{\Delta }}}_{1}={{\rm{\Delta }}}_{b}^{0}+{\alpha }_{3},{{\rm{\Delta }}}_{2}={{\rm{\Delta }}}_{b}^{0}-{\alpha }_{3}$$

For the stability of the system, it demands that all the eigenvalues of the drift matrix *J* have negative real parts. For the hybrid optomechanical system, its stability conditions can be obtained by applying the Routh-Hurwitz criteria^54^. According to stability conditions, we will discuss the results of numerical analyses of the cooling characteristics of the mechanical oscillator of the hybrid optomechanical system in the next section.

## Results and Discussions

In this section, we concentrate on the ground-state cooling of mechanical motion and the influence of the higher-order excited atomic medium on the cooling characteristics. We solve Eqs (11–14) by performing its Fourier transform to get the quantum fluctuation of the position around the steady state as $\delta q(\omega )=\chi (\omega )F(\omega )$, where *F*(*ω*) is the Fourier transform of the total force which acts on the the mechanical oscillator and *χ*(*ω*) is the effective mechanical coefficient of the mechanical oscillator. In particular, by using the effective resonance frequency *ω*_*eff*_ and effective damping rate *γ*_*eff*_ of the movable mirror, the effective mechanical coefficient is written as $\chi (\omega )={\omega }_{m}/({\omega }_{eff}^{2}-{\omega }^{2}+i\omega {\gamma }_{eff})$, where we define

15 $${\omega }_{eff}=\sqrt{{\omega }_{m}Re[\frac{A(\omega )+B(\omega )+D(\omega ){\omega }_{m}}{D(\omega )}]},$$

and

16 $${\gamma }_{eff}={\gamma }_{m}+\frac{{\omega }_{m}}{\omega }Im[\frac{A(\omega )+B(\omega )+D(\omega ){\omega }_{m}}{D(\omega )}],$$

with

$$\begin{array}{rcl}D(\omega ) & = & [{\alpha }_{2}{\beta }_{1}-{{\rm{\Delta }}}_{2}({\alpha }_{2}^{2}{\rm{\Delta }}+{\alpha }_{5}^{2}{\rm{\Delta }}-{{\rm{\Delta }}}_{1}{\beta }_{3})+(i\omega +{\gamma }_{2})\\  &  & \times \,[{\alpha }_{2}{\rm{\Delta }}({\alpha }_{6}-{\alpha }_{5})+{\alpha }_{5}{\alpha }_{6}(-i\omega +\kappa )\\  &  & +\,{\beta }_{3}(i\omega +{\gamma }_{1})+{\alpha }_{2}^{2}(-i\omega +\kappa )]+{\beta }_{2}],\\ A(\omega ) & = & -\,{\chi }_{2}[[-{\alpha }_{6}({\alpha }_{5}(i\omega +{\gamma }_{1})-{\alpha }_{2}{{\rm{\Delta }}}_{1})+(i\omega +{\gamma }_{2})({\beta }_{6}-{\alpha }_{2}^{2})\\  &  & -\,{{\rm{\Delta }}}_{2}({\alpha }_{2}{\alpha }_{5}+{{\rm{\Delta }}}_{1}(-i\omega +\kappa ))]{\chi }_{1}+{\beta }_{4}],\\  & = & {\chi }_{1}[{\beta }_{5}+[-{\alpha }_{2}[{\alpha }_{2}(i\omega +{\gamma }_{1})+{\alpha }_{6}{{\rm{\Delta }}}_{1}]+(i\omega +{\gamma }_{2})({\beta }_{6}-{\alpha }_{5}{\alpha }_{6})\\ B(\omega ) &  & +\,{{\rm{\Delta }}}_{2}[{\alpha }_{2}{\alpha }_{5}+{{\rm{\Delta }}}_{1}(i\omega -\kappa )]]{\chi }_{2}],\\ {\beta }_{1}(\omega ) & = & {\alpha }_{2}^{3}+{\alpha }_{5}(i\omega +{\gamma }_{1}){\rm{\Delta }}+{\alpha }_{6}{{\rm{\Delta }}}_{2}(-i\omega +\kappa )+{\alpha }_{2}({\alpha }_{5}{\alpha }_{6}-{{\rm{\Delta }}{\rm{\Delta }}}_{1}-{\beta }_{6}),\\ {\beta }_{2}(\omega ) & = & {\alpha }_{6}[{\alpha }_{5}^{2}{\alpha }_{6}-{\alpha }_{6}{{\rm{\Delta }}{\rm{\Delta }}}_{1}+{\alpha }_{5}({\alpha }_{2}^{2}-{\beta }_{6})-{\alpha }_{2}[(i\omega +{\gamma }_{1}){\rm{\Delta }}+{{\rm{\Delta }}}_{1}(-i\omega +\kappa )]],\\ {\beta }_{3}(\omega ) & = & {{\rm{\Delta }}}^{2}+{(i\omega -\kappa )}^{2},\\ {\beta }_{4}(\omega ) & = & [{\alpha }_{2}{\alpha }_{6}(-{\gamma }_{1}+{\gamma }_{2})-{\alpha }_{6}^{2}{{\rm{\Delta }}}_{1}-{\alpha }_{2}^{2}{{\rm{\Delta }}}_{2}+{\rm{\Delta }}{\beta }_{7}]{\chi }_{2},\\ {\beta }_{5}(\omega ) & = & [{\alpha }_{2}{\alpha }_{5}(-{\gamma }_{1}+{\gamma }_{2})+{\alpha }_{2}^{2}{{\rm{\Delta }}}_{1}+{\alpha }_{5}^{2}{{\rm{\Delta }}}_{2}-{\rm{\Delta }}{\beta }_{7}]{\chi }_{1},\\ {\beta }_{6}(\omega ) & = & (i\omega +{\gamma }_{1})(i\omega -\kappa ),\\ {\beta }_{7}(\omega ) & = & (i\omega +{\gamma }_{1})(i\omega +{\gamma }_{2})+{{\rm{\Delta }}}_{1}{{\rm{\Delta }}}_{2},\end{array}$$

In order to count the effective phonon number in the mechanical motion, we need to calculate the position and momentum noise spectrum of the mechanical oscillator. Using Eqs (11–14) and the spectrum relation ${\langle \delta q(\omega ^{\prime} )\delta q(\omega )\rangle }_{s}={S}_{q}(\omega )\delta (\omega ^{\prime} +\omega )$ ^27,55^ we can get the position noise spectrum *S*_*q*_(*ω*) of the mechanical motion as follows:

17 $${S}_{q}(\omega )=|\chi (\omega {)|}^{2}[{S}_{t}(\omega )+{S}_{a}(\omega )+{S}_{B}(\omega )],$$

where,

$$\begin{array}{rcl}{S}_{t}(\omega ) & = & \frac{{\gamma }_{m}\omega }{{\omega }_{m}}(1+\,\cot \,{\rm{h}}\frac{\hslash \omega }{2{\kappa }_{B}T}),\\ {S}_{a}(\omega ) & = & \frac{2\,\kappa }{|D(\omega {)|}^{2}}[|{h}_{1}(\omega {)|}^{2}+|{h}_{2}(\omega {)|}^{2}],\\ {S}_{B}(\omega ) & = & \frac{2{\gamma }_{b}}{|D(\omega {)|}^{2}}[|{h}_{3}(\omega {)|}^{2}+|{h}_{4}(\omega {)|}^{2}],\\ {S}_{B}(\omega ) & = & \frac{2{\gamma }_{b}}{|D(\omega {)|}^{2}}[|{h}_{3}(\omega {)|}^{2}+|{h}_{4}(\omega {)|}^{2}],\\ {h}_{1}(\omega ) & = & [{\alpha }_{2}^{2}(i\omega +{\gamma }_{1})+{\alpha }_{5}{\alpha }_{6}(i\omega +{\gamma }_{2})\\  &  & +\,{\alpha }_{2}({\alpha }_{6}{{\rm{\Delta }}}_{1}-{\alpha }_{5}{{\rm{\Delta }}}_{2})-(i\omega -\kappa ){\beta }_{7}]{\chi }_{1}+{\beta }_{4},\\ {h}_{2}(\omega ) & = & {\beta }_{5}-[{\alpha }_{5}{\alpha }_{6}(i\omega +{\gamma }_{1})+{\alpha }_{2}^{2}(i\omega +{\gamma }_{2})\\  &  & +\,{\alpha }_{2}(-{\alpha }_{6}{\Delta }_{1}+{\alpha }_{5}{{\rm{\Delta }}}_{2})-(i\omega -\kappa ){\beta }_{7}]{\chi }_{2},\\ {h}_{3}(\omega ) & = & [{\alpha }_{2}^{3}+{\alpha }_{6}\Delta (i\omega +{\gamma }_{2})+{\alpha }_{5}{{\rm{\Delta }}}_{2}(-i\omega +\kappa )\\  &  & +\,{\alpha }_{2}({\alpha }_{5}{\alpha }_{6}-{{\rm{\Delta }}{\rm{\Delta }}}_{2}){\beta }_{8}]{\chi }_{1}+[-{\alpha }_{5}({\alpha }_{6}^{2}-{{\rm{\Delta }}{\rm{\Delta }}}_{2})\\  &  & +\,{\alpha }_{6}(-{\alpha }_{2}^{2}+{\beta }_{8})+{\alpha }_{2}[(i\omega +{\gamma }_{2}){\rm{\Delta }}+{{\rm{\Delta }}}_{2}(-i\omega +\kappa )]]{\chi }_{2},\\ {h}_{4}(\omega ) & = & -\,{\beta }_{2}{\chi }_{1}-{\beta }_{1}{\chi }_{2},\\ {\beta }_{8}(\omega ) & = & (i\omega +{\gamma }_{2})(i\omega -\kappa ),\end{array}$$

Correspondingly, the momentum noise spectrum *S*_*p*_(*ω*) of the mechanical motion can be attained directly via ${S}_{p}(\omega )={(\omega /{\omega }_{m})}^{2}{S}_{q}(\omega )$.

Using the position and momentum noise spectrum of the mechanical oscillator *S*_*q*_(*ω*) and *S*_*p*_(*ω*), the effective phonon number about quantum harmonic oscillator can be defined as

18 $${N}_{f}=(\langle \delta {p}^{2}\rangle +\langle \delta {q}^{2}\rangle -\mathrm{1)/2,}$$

where $\langle \delta {p}^{2}\rangle$ and $\langle \delta {q}^{2}\rangle$ are the variances of momentum and displacement of the mechanical oscillator in the case of steady state, and are expressed as $\langle \delta {p}^{2}\rangle =\frac{1}{2\pi }{\int }_{-\infty }^{\infty }{S}_{p}(\omega )d\omega$ and $\langle \delta {q}^{2}\rangle =\frac{1}{2\pi }{\int }_{-\infty }^{\infty }{S}_{q}(\omega )d\omega$, respectively^37^.

In the following, we will apply the graph to describe the characteristics of ground-state cooling of the quantum harmonic oscillator in this system. We choose some accessible parameters in our optomechanical system, i.e, the high-excitation limit for the atoms is considered with ${b}_{s}=-0.2+0.4i$. The other parameter values we select are *ω*_*m*_ = 2*π* × 9.47 × 10^5^ *Hz*, *κ* = 2*π* × 2.15 × 10^5^ *Hz*, Δ = *ω*_*m*_, *N* = 10^6^, *G* = *g* × *N* ^0.5^, *λ* = 791 *nm*, *L* = 0.001 *m*, *M* = 5 × 10^−12^ *kg*, *γ*_*m*_ = *ω*_*m*_/6700, *γ*_*b*_ = 2*π* × 7500 *Hz*.

**Figure 2:**
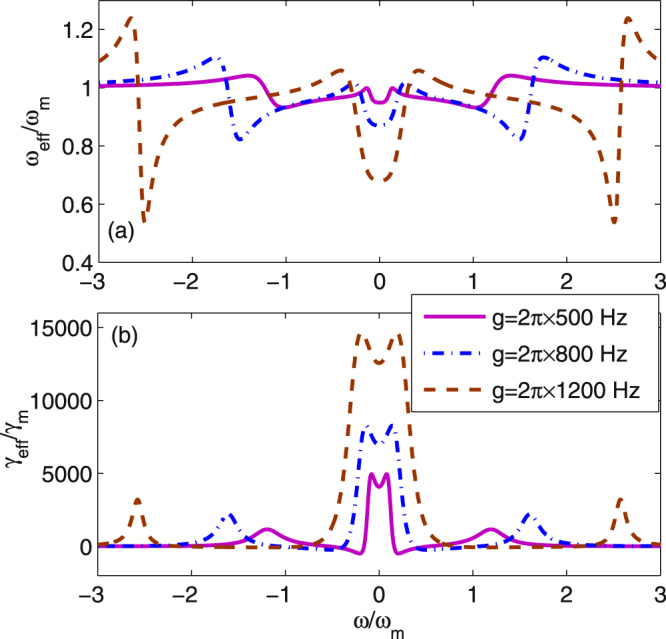
The normalized effective oscillation frequency *ω*_*eff*_/*ω*_*m*_ and the normalized effective damping rate ${\gamma }_{eff}/{\omega }_{m}$ are plotted as a function of the normalized frequency *ω*/*ω*_*m*_ with different coupling strength between the atom-field and the cavity field.

As shown in Fig. 2(a), we plot the normalized effective oscillation frequency *ω*_*eff*_/*ω*_*m*_ as a function about the normalized frequency *ω*/*ω*_*m*_ with different coupling strength between the atomic ensemble and the cavity field when ${b}_{s}=-0.2+0.4i$. In this case, ${\varepsilon }_{c}=856.16\,\kappa$ and ${{\rm{\Delta }}}_{b}=5.2\,\kappa$. From Fig. 2(a), it is shown that the frequency of the movable mirror is not changed significantly with a small atom-field coupling strength, i.e., $g=2\pi \times 500\,Hz$. The change of the effective frequency becomes large with increasing the coupling strength *g*, which means the optical spring effect of the system is significant. The normalized *γ*_*eff/*_ *γ*_*m*_ as a function of the normalized frequency *ω*/*ω*_*m*_ for the different coupling strength *g* is shown in Fig. 2(b). From Fig. 2(b), it is seen that the effective damping rate increases significantly with increasing the coupling strength, that is, the effective damping rate can be improved by adding the coupling strength between atom and cavity. It is known to all that the effective damping rate is a basic criterion to determine whether the cooling of a mechanical resonator is close to the quantum ground state. Thus a large coupling strength is favorable to enhancing the effective damping rate. In addition, the cooling of the ground state of a mechanical oscillator requires the effective phonon number ${N}_{f} < 1$, which means the initial mean-thermal excitation number $n={[exp(\hslash {\omega }_{m}/{\kappa }_{B}T)-\mathrm{1]}}^{-1}$ is not an excessive quantity, which is possibly realized when *ω*_*m*_ is sufficiently large and the temperature is low enough.

**Figure 3:**
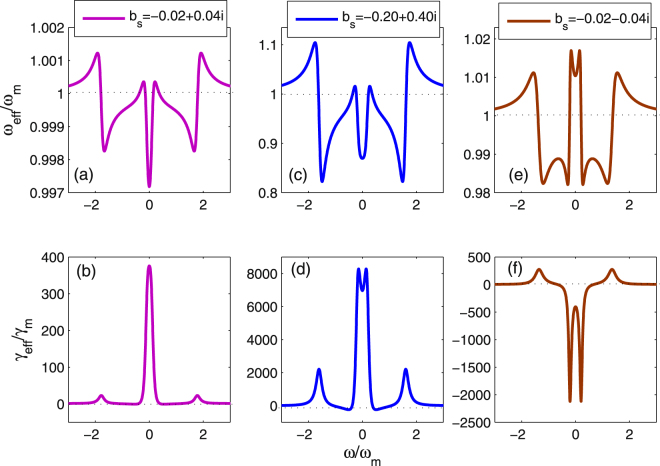
The normalized effective oscillation frequency *ω*_*eff*_/*ω*_*m*_ and the normalized effective damping rate ${\gamma }_{eff}/{\omega }_{m}$ are plotted as a function of the normalized frequency *ω*/*ω*_*m*_ with different *b*_*s*_’s. Here, $g=2\pi \times 800\,Hz$, and other parameter values are same as in Fig. 2.

We now discuss the effect of the excitation number of the atomic ensemble on *ω*_*eff*_ and *γ*_*eff*_. In Fig. 3, we show the normalized effective oscillation frequency *ω*_*eff*_/*ω*_*m*_ and the normalized effective oscillation damping rate *γ*_*eff/*_ *γ*_*m*_ as a function of the normalized frequency *ω*/*ω*_*m*_ with different values of *b*_*s*_. We consider the three cases with ${b}_{s}=-0.02+0.04i$, ${b}_{s}=-0.20+0.40i$ and ${b}_{s}=-0.02-0.04i$. Correspondingly, the driving strength are ${\varepsilon }_{c}=89.107\,\kappa ,\,865.16\,\kappa$ and $236.789\,\kappa$, respectively; the atomic detuning are ${{\rm{\Delta }}}_{b}=8.6\,\kappa ,\,5.2\,\kappa$ and 0.36 *k*, respectively. From Fig. 3(a,c) and Fig. 3(b,d), it is seen that the excitation number of the atoms influences strongly the optical spring effect and the effective damping rate. For example, the *γ*_*eff*_ increases with increasing excitation number. Further, when the excitation number is large, i.e., ${b}_{s}=-0.2+0.40i$, the effective damping rate is positive. However, when ${b}_{s}=-0.02-0.04i$, the effective damping rate becomes negative, which means that the heating process appears. [Fig. 3(f)]. In order to achieve the ground-state cooling of the mechanical oscillator, we should select properly the steady-state of the atomic ensemble. From Figs 2 and 3, we find that the excitation number and the atom-field coupling strength play a similar role for enhancing the effective damping rate in this system.

**Figure 4:**
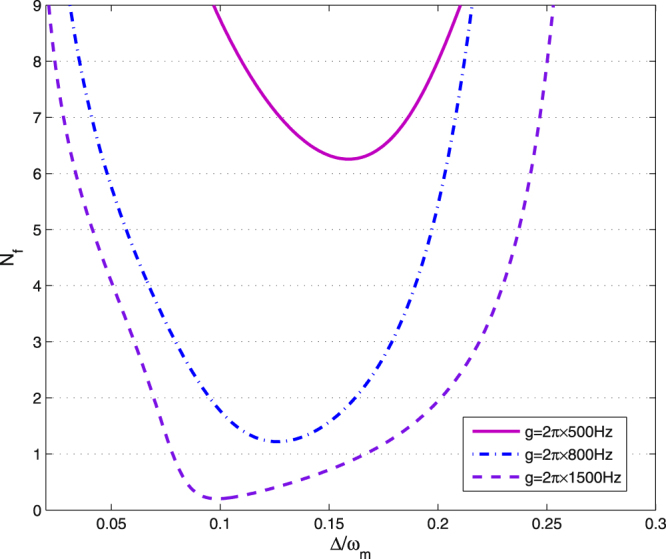
The effective phonon number *N*_*f*_ is plotted as a function of the dimensionless effective cavity detuning ${\rm{\Delta }}/{\omega }_{m}$ with different *g*. Other parameter values are same as in Fig. 2.

In Fig. 4, we show the effective phonon number *N*_*f*_ as a function of the dimensionless effective cavity detuning ${\rm{\Delta }}/{\omega }_{m}$ with different *g*. It is clear that the effective phonon number is always larger than 1 with a small coupling strength, i.e., $g=2\pi \times 500\,Hz$. Thus, the ground state of the mechanical resonator can not be achieved. Obviously, the minimum of the effective phonon number decreases with increasing *g*. For example, when $g=2\pi \times 1500\,Hz$, the minimum effective phonon number is smaller than 1. Therefore, the ground-state cooling of the mechanical oscillator can be obtained with a large coupling strength *g*, which results from a large optomechanical coupling between the cavity field and the mechanical oscillator.

**Figure 5:**
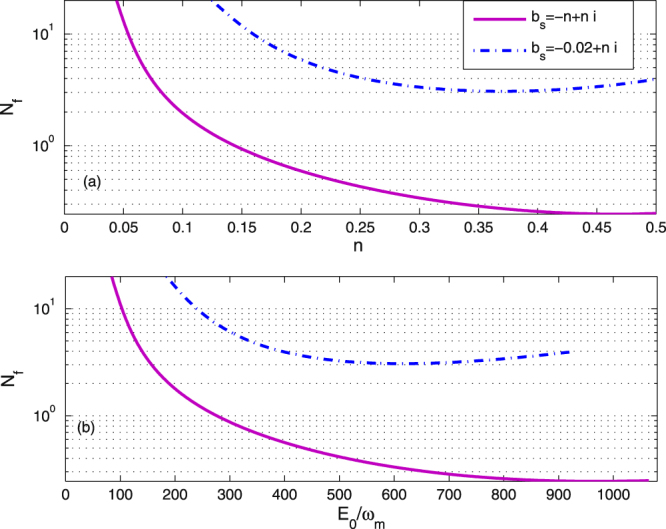
The effective phonon number *N*_*f*_ is plotted as a function of *n* and ${E}_{0}/{\omega }_{m}$. Other parameter values are same as in Fig. 2.

We can study the effect of the excitation number and the driven strength ${E}_{0}/{\omega }_{m}$ on the *N*_*f*_. In Fig. 5, we show the effective phonon number *N*_*f*_ as a function of the imaginary part *n* with $m=-n$ and $m=-0.02$. It is seen from Fig. 5(a) that the effective phonon number *N*_*f*_ can be small than 1 and decreases with increasing *n* as $m=-n$. In contrast, in the case of fixed value, i.e., $m=-0.02$, the effective phonon number *N*_*f*_ can not be smaller than 1 with different imaginary part *n*. Therefore, the identical real and imaginary parts for the steady-state values of the atomic ensemble can be applied to help achieve ground-state cooling of the mechanical oscillator. It is clear from Fig. 5(b) that the effective phonon number *N*_*f*_ decreases with increasing driven strength ${E}_{0}/{\omega }_{m}$ at $m=-n$, i.e., *n* = 0.5, and ${b}_{s}=-0.5+0.5i$. When $m\ne n$, i.e., ${b}_{s}=-0.02+0.5i$, the effective phonon number *N*_*f*_ is always larger than 1 regardless of the driving strength *E*_0_. In particular, we can not achieve the ground-state of the mechanical resonator by increasing the driving strength *E*_0_ when the atomic ensemble satisfies the low-excitation condition.

**Figure 6:**
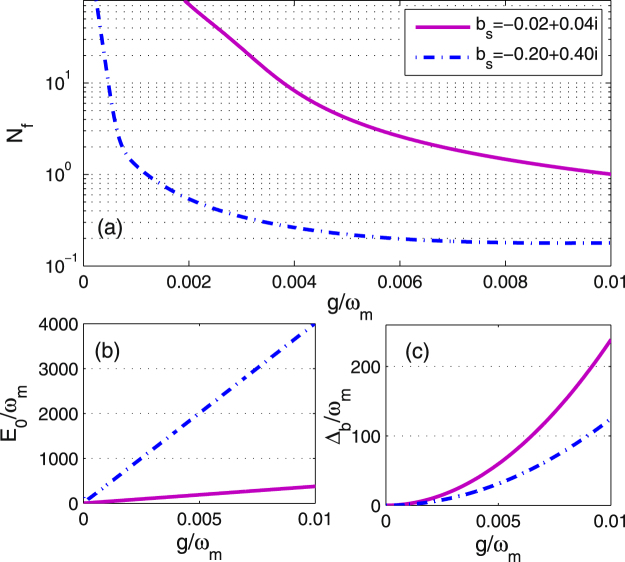
The effective phonon number *N*_*f*_ and the corresponding effective driving strength ${E}_{0}/{\omega }_{m}$ and the dimensionless effective atomic detuning ${{\rm{\Delta }}}_{b}/{\omega }_{m}$ are plotted as a function of $g/{\omega }_{m}$ in the absence and presence of higher order atomic excitation. Other parameter values are same as in Fig. 2.

In Fig. 6(a), we plot the effective phonon number *N*_*f*_ as a function of the coupling strength $g/{\omega }_{m}$ with different *b*_*s*_, i.e., ${b}_{s}=-0.02+0.04i$, and ${b}_{s}=-0.20+0.40i$. The aim of Fig. 6(a) is to show the significant role of the atomic ensemble and atom-field coupling for the cooling of the movable mirror in the optomechanical system. From Fig. 6(a), it is seen that the effective phonon number is smaller than 1 as $g > 0.001{\omega }_{m}$ with ${b}_{s}=-0.20+0.40i$, which means that the atom-field coupling strength enhances the cooling process of the system. Figure 6(b) and (c) show the relationships between ${E}_{0}/{\omega }_{m}$, ${{\rm{\Delta }}}_{b}/{\omega }_{m}$ and $g/{\omega }_{m}$ with different *b*_*s*_. It is clearly seen that in presence of the higher-order excitation of atom, i.e., ${b}_{s}=-0.20+0.40i$, a large driving of the cavity but a relatively small atom-field detuning can be used for achieving the ground-state cooling of the mechanical oscillator. The present model has a potential application for studying the influence of the atom medium on the dynamics of a typical optomechanical system and probing the ground-state cooling of a mechanical motion.

## Conclusion

In summary, we have investigated the ground-state cooling of a quantum harmonic oscillator in a hybrid optomechanical system. In our physical model, we consider three kinds of couplings: the coupling between the cavity field and the mechanical oscillator, the coupling of the cavity field with the driving laser field, and the coupling of the cavity interacting with the atomic ensembles. In addition, the atomic ensemble consisting of many two-level atoms is in the case of higher-order excitation. For the high quality cavity, strong coupling between atoms and cavity field can be achieved experimentally. Furthermore, we use the coupling laser to drive the cavity field, and the coupling between cavity and laser field can increase the number of photon in the cavity, which can enhance the interaction between atomic ensemble and cavity field. So in this paper, we consider the case in which the atomic ensemble is in higher excitation, that is, the excitation probability of a single atom is very large. In the case of multi-coupling mechanism, we build the quantum Langevin equation of the system and obtain its dynamical properties under the condition of steady state. By performing the Fourier transform on quantum fluctuation of mechanical quantities, we derive the effective damping rate and the effective frequency of the mechanical oscillator. We numerically analyze the influence the of coupling strength *g* and the excitation number *b*_*s*_ on the effective damping and effective frequency of the mechanical oscillator. Furthermore, we also discuss in detail the cooling characteristics of the quantum harmonic oscillator by calculating the effective phonon number with different coupling strength *g* and the atomic excitation number *b*_*s*_. Our numerical results show that if the coupling strength *g* and the atomic excitation number *b*_*s*_ are selected properly, the ground-state cooling of the mechanical oscillator can be achieved. In this hybrid optomechanical system, our studied about the influence of the atomic ensemble with higher-order excitation on the ground-state cooling of a mechanical oscillator have potential application in some fields, such as precision measurement, quantum squeezing and high precision spectrum, and son on.
